# Carbohydrate Sensing Through the Transcription Factor ChREBP

**DOI:** 10.3389/fgene.2019.00472

**Published:** 2019-06-04

**Authors:** Paula Ortega-Prieto, Catherine Postic

**Affiliations:** Université de Paris, Institut Cochin, CNRS, INSERM, Paris, France

**Keywords:** ChREBP, carbohydrate sensing, transcriptional regulation, metabolism, insulin sensitivity

## Abstract

Carbohydrate response element binding protein (ChREBP) is a carbohydrate-signaling transcription factor that in the past years has emerged as a central metabolic regulator. ChREBP expression is mostly abundant in active sites of *de novo* lipogenesis including liver and white and brown adipose tissues. ChREBP is also expressed in pancreatic islets, small intestine and to a lesser extent in the kidney and the brain. In response to glucose, ChREBP undergoes several post-translational modifications (PTMs) (phosphorylation, acetylation and/or *O*-GlcNAcylation) that will either modulate its cellular location, stability and/or its transcriptional activity. ChREBPβ is a shorter isoform of ChREBP that was first described in adipose tissue and later found to be expressed in other sites including liver and pancreatic β cells. ChREBPβ lacks an important regulatory inhibitory domain, known as LID (low glucose inhibitory domain), in its N-terminal domain and is therefore reported as a highly active isoform. In this review, we recapitulate a recent progress concerning the mechanisms governing the activity of the ChREBP isoforms, including PTMs, partners/cofactors as well as novel metabolic pathways regulated by ChREBP in key metabolic tissues, by discussing phenotypes associated with tissue-specific deletion of ChREBP in knockout mice.

## Introduction

Increased consumption of simple sugars such as sucrose and high-fructose corn syrup in recent years has led to an increased risk of metabolic diseases such as obesity, dyslipidemia, type 2 diabetes and/or nonalcoholic fatty liver disease (NAFLD). The liver is the principal organ responsible for the conversion of excess dietary carbohydrates into fat. The resulting triglycerides (TG) can be packed into very low density lipoproteins (VLDL) and either secreted into the circulation, stored as lipid droplets, or metabolized through the beta-oxidation pathway. Insulin secreted in response to elevated blood glucose, stimulates the expression of genes of *de novo* fatty acid synthesis (lipogenesis) through the transcription factor sterol regulatory element binding protein-1c (SREBP-1c) (Foretz et al., 1999). SREBP-1c acts in synergy with another transcription factor called carbohydrate response element binding protein (ChREBP), which mediates the response to dietary carbohydrates. The ChREBP protein structure contains a low glucose inhibitory domain (LID) and a glucose-response activation conserved element (GRACE) located in its N-terminus (Li et al., 2006). Activation of the GRACE domain by glucose metabolites promotes ChREBP transcriptional activity and binding to a highly conserved sequence called carbohydrate response element (ChoRE). ChoRE is present on the promoters of ChREBP target genes, which encode key enzymes of *de novo* lipogenesis including L-pyruvate kinase (*L-pk*), a rate-limiting enzyme in glycolysis, fatty acid synthase (*Fas*), acetyl-CoA carboxylase (*Acc*) and stearoyl-CoA desaturase (*Scd1*) (Kawaguchi et al., 2001). A recent study reported the interdependence between ChREBP (activated by glucose) and SREBP-1c (activated by insulin) for the full induction of glycolytic and lipogenic gene expression in liver (Linden et al., 2018). Viral re-establishment of the nuclear active form of SREBP-1c in the liver of ChREBP-deficient mice (ChREBP^KO^) normalized lipogenic gene expression, while having no effect on rescuing glycolytic gene expression. The mirror experiment, in which ChREBP expression was induced in the liver of SREBP-1c knockout mice, rescued glycolytic gene expression but surprisingly not lipogenic gene expression, despite the well-known role of ChREBP in the control of fatty acid synthesis genes. Nevertheless, this study suggests the importance of the dual action of ChREBP and SREBP-1c in the control genes involved in the regulation of fatty acid synthesis (Linden et al., 2018).

ChREBP is highly enriched in the liver and has been studied as a master regulator of lipid metabolism (Iizuka et al., 2004; Osorio et al., 2016). ChREBP is also significantly expressed in pancreatic islets, small intestine, skeletal muscle and to a lesser extent in the kidney and the brain (see Richards et al., 2017 for review). Interestingly, another isoform of ChREBP, ChREBPβ, originating from an alternative first exon promoter, was first identified in adipose tissue (Herman et al., 2012) and later described in other cell types (see Abdul-Wahed et al., 2017 for review). As we will discuss, ChREBPβ is described as a constitutively active isoform. It is hoped that future work will address the respective roles of ChREBP and ChREBPβ in the regulation of glucose and lipid metabolism as well as identify their specific and/or overlapping targets.

## ChRebp Structure and Regulation Via the Lid/Grace Domains

**FIGURE 1:**
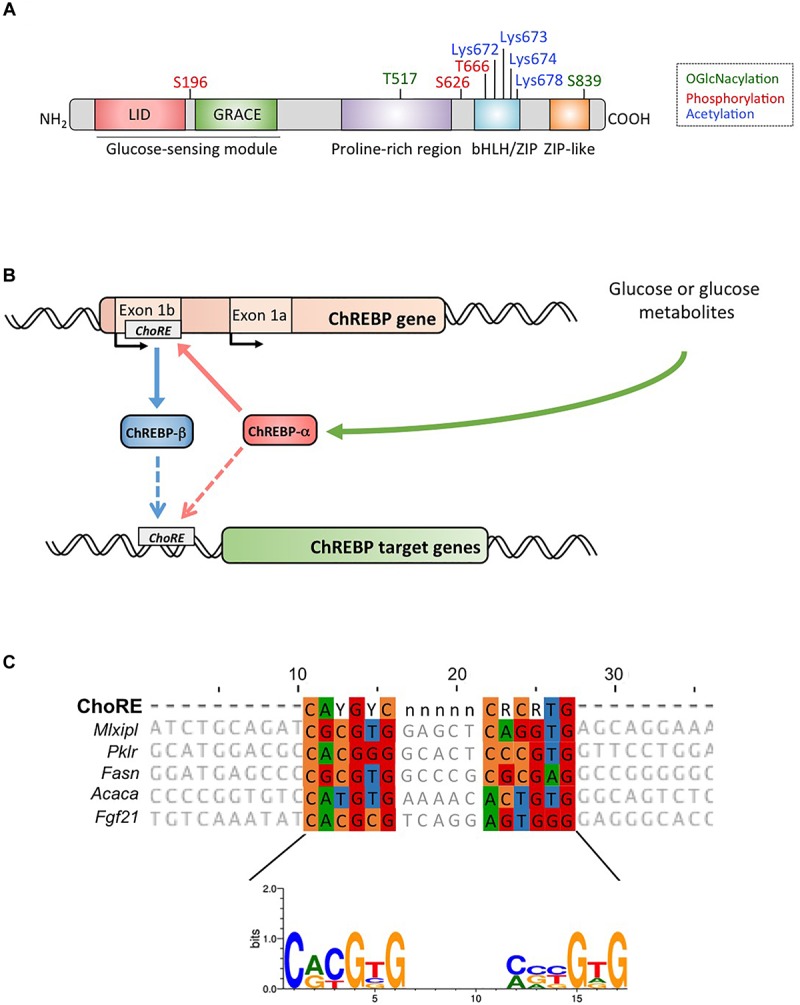
**(A)** Structure of carbohydrate response element binding protein α (ChREBPα). ChREBPα is composed of 864 amino acids and contains several regulatory domains. At the N-terminus the protein contains a glucose-sensing module composed of the low glucose inhibitory domain (LID) and the glucose activated conserved element (GRACE). The protein also contains a polyproline-rich, a bHLH/LZ and a leucine-zipper-like (Zip-like) domain located at the C-terminus. Post-translational modifications are indicated in their respective residues, phosphorylation (red), acetylation (blue) and the recently identified *O*-GlcNAcylations (green). **(B)** Gene structure of the ChREBP gene and generation of the two ChREBP isoforms α and β. ChREBPβ is transcribed from an alternative first exon promoter 1b. This transcript is translated from exon 4 generating a shorter protein of 687 amino acids in which the two NES, the NLS and the LID domain are missing. The ChREBPβ isoform has been suggested to be directly regulated by ChREBPα since a ChoRE sequence was identified in the exon promoter 1b. Whether both ChREBP α and β isoforms both bind to the ChoRE is currently not known. Figure adapted from Herman et al. (2012). **(C)** Multi-alignment of ChoRE consensus sequences presents in several ChREBP target gene promoters. Nucleotides-based alignment is presented on the top of the figure together with the consensus sequence ChoRE described in Poungvarin et al. (2015). The logo corresponding to the consensus sequence associated to this particular alignment is also represented.

ChREBP belongs to the Mondo family of bHLH/Zip transcription factors. The N-terminus domain (1-251 residues) contains two nuclear export signal (NES) and a nuclear localization signal (NLS) regulating subcellular localization by interacting with chromosome region maintenance 1 (CRM1) also referred to as exportin 1 and/or 14-3-3 proteins (Sakiyama et al., 2008). The C-terminus region contains a polyproline domain, a bHLH/LZ domain (660-737 residues) and a leucine zipper-like domain (Zip-like, 807-847 residues) that are associated with co-factors and DNA binding (Yamashita et al., 2001; Fukasawa et al., 2010; Ge et al., 2012). Localization and transcriptional activation of ChREBP are determined by nutrient availability. Glucose-mediated regulation of ChREBP occurs mostly at the level of the glucose-sensing module (GSM) or mondo conserved region (MCR), which is composed of the LID and the GRACE domains, as mentioned in the introduction (Figure 1A>; Li et al., 2006; Singh and Irwin, 2016). In 2012, Herman et al. (2012) described another ChREBP isoform, ChREBPβ, that is transcribed from an alternative first exon promoter 1b to exon 2 (Figure 1B>). This transcript is translated from exon 4, generating a shorter protein of 687 amino acids (the full length ChREBP isoform, renamed α, contains 864 amino acids, called ChREBP in the manuscript) in which the two NES, NLS and the LID domain are missing. ChREBPβ is highly active in white adipose tissue in a GLUT-4 dependent manner and is suggested to be directly regulated by ChREBPα since a ChoRE sequence was identified in the exon promoter 1β (Herman et al., 2012; Figure 1B>). The regulation of ChREBPβ by ChREBPα suggests the existence of a feed-forward loop that potentially exacerbates the response to glucose under hyperglycemic conditions. However, the regulatory mechanism(s) of the ChREBPβ isoform, and more importantly its specific function, need to be elucidated.

## Activation of ChRebp by Glucose Metabolites

Under fasting conditions, the glucagon-dependent activation of protein kinase A (PKA) (Kawaguchi et al., 2002) phosphorylates ChREBP on residues Ser196 and Thr666, leading to ChREBP binding to the protein 14-3-3 and its retention in the cytosol (Kawaguchi et al., 2001, 2002; Davies et al., 2008). AMP activated protein kinase (AMPK), a central cellular energy sensor, also phosphorylates ChREBP on residue Ser568, which in turn decreases binding of ChREBP to promoters of its target genes (Kawaguchi et al., 2002; Sato et al., 2016). It was demonstrated that metabolites generated during fasting, such as AMP and ketone bodies produced from fatty acid oxidation, play an allosteric inhibitory role by altering ChREBP and 14-3-3 protein affinity, enhancing complex stabilization and favoring cytosolic retention (Sakiyama et al., 2008; Nakagawa et al., 2013; Sato et al., 2016). In response to carbohydrates, ChREBP is regulated at the transcriptional, translational and post-translational levels. Increased glucose concentrations after a meal promote the synthesis of intermediate metabolites such as xylulose-5-phosphate (X5P), initially proposed as an activator of protein phosphatase 2A (PP2A) (Kawaguchi et al., 2001; Kabashima et al., 2003). PP2A was previously described to dephosphorylate ChREBP at the Ser196 residue allowing its translocation to the nucleus where it is be further dephosphorylated in a X5P- and PP2A-dependent manner (on Thr666 and Ser568). However, this model was challenged over the years and other metabolites such as glucose-6-phosphate (G6P) were proposed to be potential activators of ChREBP translocation/activity (Dentin et al., 2012). McFerrin et al. (2012) identified a putative motif for G6P binding (253-SDTLFT-258) on the GRACE domain, which is also conserved in MondoA, a ChREBP/MondoB ortholog (see Richards et al., 2017 for review). According to this hypothesis, G6P could promote an allosteric conformation change that induces an open conformation for ChREBP, facilitating the interaction with co-factors and subsequent translocation to the nucleus (McFerrin et al., 2012).

Within the nucleus, ChREBP can be modified through *O-*GlcNAcylation, a post-translational modification dependent on glucose metabolism, and identified to be important for ChREBP transcriptional activity (Guinez et al., 2010). *O-*GlcNAcylation occurs on serine and threonine residues through the activity of *O*-GlcNAc transferase (OGT), an enzyme that adds *N*-acetylglucosamine (GlcNAc) residues to target proteins thereby modifying their activity, stability and/or subcellular location. Yang et al. (2017) recently revealed several ChREBP residues modified by *O*-GlcNacylation. Mutations of these residues at the bHLH/ZIP and dimerization and cytoplasmic location domain (DCD) domains have allowed for the identification of Thr517 and Ser839 as essential sites for the glucose-dependent activation of ChREBP (Figure 1A>). ChREBP can also be modified by acetylation via the histone acetyltransferase activity of p300 (Bricambert et al., 2010). Glucose-activated p300 acetylates ChREBP on Lys672 and increases its transcriptional activity by enhancing its recruitment to the ChoRE sequence, which’s optimal consensus binding sequence is CAYGYCnnnnnCRCRTG (Figure 1C>). Poungvarin et al. (2015) analyzed ChREBP binding sites by ChIP-seq in liver and white adipose tissue of mice re-fed with a high-carbohydrate, fat-free diet. They reported that ChREBP binding is enriched in pathways involved in insulin signaling, adherent junctions and cancer, suggesting a novel involvement of ChREBP in tumorigenesis and cancer progression. Further, a recent study reported the importance of ChREBP in hepatocellular carcinoma (HCC) (Ribback et al., 2017). The authors found that genetic deletion of ChREBP (in ChREBP^KO^ mice) impaired hepatocarcinogenesis driven by protein kinase B/Akt overexpression in mice. Furthermore, siRNA-mediated inhibition of ChREBP in mouse and/or human HCC cells resulted in decreased proliferation and apoptosis.

## ChRebp Co-Factors and Partners

Several co-factors and/or partners of ChREBP were identified over the past years (see Richards et al., 2017 for review). Max like protein x (Mlx), a bHLH/LZ transcription factor, was the first identified as a common binding partner of the Mondo family (Stoeckman et al., 2004). Dimerization of ChREBP with Mlx is required for both nuclear translocation in response to glucose and binding to ChoRE elements. Nuclear receptors such as hepatocyte nuclear factor 4α (HNF4α) and farnesoid X receptor (FXR) were also described as ChREBP partners. HNF4α physically interacts with ChREBP by binding to the direct repeat-1 (DR-1) region on the promoter of ChREBP target genes (Adamson et al., 2006; Meng et al., 2016). Moreover, the p300/CBP transcriptional co-activator proteins were shown to stabilize the ChREBP/HNF4α complex (Burke et al., 2009). The p300/CBP transcriptional co-activator proteins play a central role in coordinating and integrating multiple signal-dependent events with the transcription apparatus. Another key property of p300/CBP is the presence of histone acetyltransferase (HAT) activity, which endows p300/CBP with the capacity to influence chromatin activity by modulating nucleosomal histones. In human hepatocytes, FXR binding to the ChREBP-HNF4α complex triggers the release of ChREBP from CBP/p300, leading to the recruitment of the histone deacetylase SMRT on the *Lpk* promoter, thereby acting as a co-repressor of ChREBP transcriptional activity (Caron et al., 2013). In addition, CBP/p300 HAT activity modifies ChREBP on Lys 672, leading to its transcriptional activation in response to glucose (Bricambert et al., 2010).

Bricambert et al. (2018) recently identified the histone demethylase plant homeodomain finger 2 (Phf2), which belongs to the histone lysine demethylase (KDM7) family, as a novel co-factor of ChREBP. Interaction between Phf2 and ChREBP enhances ChREBP transcriptional activation by erasing H3K9 methyl-marks on the promoter of its target genes. Interestingly, specific co-recruitment of Phf2 and ChREBP to the promoter of nuclear factor erythroid 2 like 2 (Nrf2) contributes to the protective effect of Phf2 against increased reactive oxygen species (ROS) and NAFLD progression in the context of hyperglycemia (Bricambert et al., 2018).

## Role of ChRebp in Carbohydrate Metabolism and Hepatokine Production

### ChREBP as Regulator of Hepatic Fatty Acid Synthesis and VLDL Secretion

Non-alcoholic fatty liver disease is a hallmark of metabolic syndrome, and studies in humans reveal that *de novo* lipogenesis contributes to about 25% of total liver lipids in patients with NAFLD (Donnelly et al., 2005). In insulin resistant states, hyperglycemia and hyperinsulinemia enhance lipogenesis partly through the activation of ChREBP and SREBP-1c. ChREBP inhibition in liver of obese and insulin resistant *ob/ob* mice, through RNAi or genetic ablation leads to reversal of hepatic steatosis (Dentin et al., 2006; Iizuka et al., 2006). Altered secretion of VLDL by the liver also contributes to the pathogenesis of NAFLD. Microsomal triglyceride transfer protein (MTTP) is the protein in charge of assembly and secreting apolipoprotein B-containing lipoproteins. Deficiency of MTTP in mice and humans causes hypolipidemia and fatty liver. Regulation of this protein has been associated with a few highly conserved cis-elements in its promoter including critical positive [HNF1, HNF4, DR-1 and forkhead box (FOX)] and negative [regulatory sterol and insulin response elements (SRE/IRE)] regulatory domains (Cuchel et al., 2013; Hussain et al., 2011). Recently, ChREBP was pointed out as a potential regulator of MTTP since lack of functional ChREBP in liver suppresses *Mttp* expression and VLDL assembly and secretion (Niwa et al., 2018). However, since no ChoRE could be clearly identified on the *Mttp* promoter, further analysis will be needed to identify the mechanism with which ChREBP regulates *Mttp*.

### Regulation of Fructose Metabolism by ChREBP in Liver and Intestine

**FIGURE 2:**
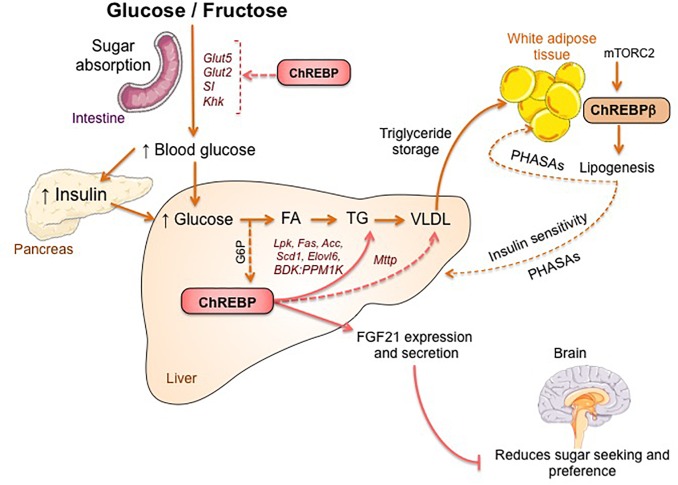
ChREBP regulates multiples signaling/metabolic pathways in response to glucose and fructose. ChREBP is expressed in several tissues including intestine, liver and white adipose tissue. In these cell types, in response to glucose and/or fructose ChREBP is activated and induces specific genic program as indicated on the figure. In intestine, stimulation of SI, Glut5, Glut2 and Ketohexokinase (Khk) expression by ChREBP (either directly or indirectly) was described to improve sucrose tolerance and fructose absorption. In liver, ChREBP is a key modulator of glycolytic, lipogenic and microsomal triglyceride transfer protein (Mttp) gene expression, thereby controlling both fatty acid accumulation and VLDL export from the liver. ChREBP is also regulates the production of hepatokines such as fibroblast growth factor 21 (FGF21). This liver-to-brain axis expands liver ChREBP function from a hepatic regulator to a systemic modulator affecting not only substrate handling in liver but also nutrient preference. ChREBP activation in white adipose tissue is linked to improved metabolic homeostasis by producing protective circulating signals. A novel class of mammalian lipids characterized by a branched ester linkage between a fatty acid and a hydroxy-fatty acid (palmitic acid hydroxyl stearic acid) was reported to exert beneficial effects on glucose homeostasis through direct and incretin-mediated modulation of β cell function, enhanced adipose glucose uptake and reduced inflammation. Interestingly, mTORC2 was recently identified as a novel regulator of ChREBPβ isoform in adipose cells.

The link between ChREBP and fructose metabolism was first evidenced by the phenotypic analysis of ChREBP knockout mice (ChREBP^KO^ mice). ChREBP^KO^ mice were reported to die within several days of high-fructose diet (HFrD) feeding (Iizuka et al., 2004). This major intolerance to fructose was attributed to the reduction in the expression of fructokinase and triose kinase, two enzymes required for fructose metabolism (Iizuka et al., 2004). Kim et al. (2016) later reported the importance of ChREBP for the efficient conversion of fructose to glucose in liver and whole-body fructose clearance but also, under ingestion of fructose, ChREBP could contribute to hyperglycemia by directly trans-activating *G6pc* expression, a key gene of gluconeogenesis. This effect could lead to a vicious cycle in which fructose consumption exacerbates glucose production though ChREBP activity (Kim et al., 2016). The following year, the study by Zhang et al. (2017) reported that ChREBP^KO^ mice fed with HFrD develop severe liver injury due to over-activation of endoplasmic reticulum stress and CCAAT-enhancer-binding protein homologous protein (CHOP)-mediated hepatocyte apoptosis. Apoptosis in hepatocytes in these mice was most likely linked to increased cholesterol biosynthesis since inhibition of this pathway via HMG-CoA reductase (HMGCR) or SREBP2 inhibition rescued ChREBP^KO^ mice from HFrD-induced liver injury. A lack of ChREBP was also recently associated with a dysregulation of sucrose and fructose metabolism leading to sugar intolerance and malabsorption in mice (Kato et al., 2018). These effects were associated with decreased expression of intestinal sucrose-isomaltase (SI), which digests sucrose in glucose and fructose, the glucose transporters 5 (Glut5) and 2 (Glut2) and the ketohexokinase (Khk) enzyme, which regulates fructolysis (Figure 2>). Dysregulation of these enzymes may lead to the accumulation of undigested sucrose and fructose with potential repercussions in gut microbiota composition. The comparison between ChREBP^KO^ and liver-specific ChREBP knockout (ChREBP^LiverKO^) mice fed with HFrD had previously revealed that hepatic ChREBP deficiency alone does not lead to fructose intolerance but that ChREBP deficiency in the small intestine is most likely responsible for the impairment in fructose tolerance observed in these mice (Kim et al., 2017). Altogether, these studies underline the importance of ChREBP in the regulation of fructose metabolism and underscore the need for a better understanding of its role and regulation in the small intestine.

### Regulation of the BDK:PPM1K Axis in Liver

The first committed step of branched-chain amino acid (BCAAs) catabolism is regulated by the branched-chain ketoacid dehydrogenase (BCKDH) complex which is controlled by two enzymes, the branched-chain alpha-keto acid dehydrogenase kinase (BDK) and the protein phosphatase, Mg^2+^/Mn2^2+^ dependent 1K (PPM1K). White et al. (2018) recently associated ChREBP with the upregulation of BDK and down-regulation of PPM1K in liver and identified a conserved ChoRE motif in the promoter of both of these genes. A positive correlation between the expression of BDK and other typical ChREBP target genes (*Fasn*, *Pklr*, ChREBPβ) was observed in livers of rats fed with a high glucose or fructose diet. At the physiological level, increase in the BDK:PPM1K ratio led to the phosphorylation and activation of ATP-citrate lyase (ACLY), thereby stimulating *de novo* lipogenesis. These findings reveal that BDK and PPM1K may be novel lipogenesis-activating genes regulated by ChREBPβ. Given their role in the regulation of lipid, glucose and amino acid metabolism, BDK and PPM1K could be considered as potential therapeutic targets in the liver in the near future (White et al., 2018).

### ChREBP Is Required for the Glucose-Mediated Regulation of FGF21

ChREBP was recently associated to the production and secretion of hepatokines such as Fibroblast growth factor 21 (FGF21) (Iizuka et al., 2009; Dushay et al., 2015, Iroz et al., 2017). FGF21 is a metabolic hormone synthetized by the liver with multiple beneficial effects in peripheral tissues (Kharitonenkov et al., 2005; Badman et al., 2007; Markan et al., 2014). Until recently, FGF21 was considered as a fasting hormone that enhances fatty acid oxidation, ketogenesis and lipolysis under the transcriptional control of peroxisome proliferator activated receptor α (PPARα) (Inagaki et al., 2007). A ChoRE on the *Fgf21* promoter has been previously identified in both mice (-74 to -52 bp) and humans (-380 to -366 bp) (Iizuka et al., 2009) but functional studies have been lacking until recently. Consumption of glucose and fructose was reported to lead to a rapid elevation of FGF21 levels in healthy volunteers and metabolic syndrome patients (Dushay et al., 2015). Additional studies also reported a mechanistic link between ChREBP-derived FGF21 and macronutrient preference through a liver-brain axis (Talukdar et al., 2016; von Holstein-Rathlou et al., 2016). This liver-to-brain axis expands ChREBP function from a hepatic metabolic regulator to a systemic modulator, affecting not only liver substrate handling but also global feeding behavior (Abdul-Wahed et al., 2017).

## Role of ChRebp in the Inter-Organ Network That Controls Energy Homeostasis

### Role of Hepatic ChREBP in the Control of Insulin Sensitivity Balance

Our laboratory previously reported that ChREBP acts as a key modulator of hepatic fatty acid composition and insulin sensitivity in the context of non-alcoholic and alcoholic liver diseases (see Abdul-Wahed et al., 2017 for review). Mice overexpressing ChREBP developed greater hepatic steatosis than controls, but interestingly stayed free of metabolic complications and did not develop insulin resistance. Lipidomic analysis has revealed that ChREBP-mediated steatosis is associated with a decrease in saturated fatty acids and an increase in monounsaturated fatty acids, the latter which have been shown to be associated with ChREBP-mediated beneficial effects on insulin sensitivity (Benhamed et al., 2012). These results demonstrate the role of ChREBP in lipid partitioning and suggest that specific lipid species, when present in the proper location and time, may trigger signals that modulate adaptation to metabolic stress (Benhamed et al., 2012; Bricambert et al., 2018). Interestingly, Jois et al. (2017) also suggested a protective role for hepatic ChREBP regarding whole body glucose homeostasis and insulin sensitivity. ChREBP^LiverKO^ mice exhibit worsened glucose tolerance, while protected from hepatic steatosis. Hepatic ChREBP deletion also resulted in gene expression changes in white and brown adipose tissues, suggesting inter-tissue communication. The contribution of ChREBP to whole body energy balance may therefore rely on its regulation of lipid species and/or hepatokine production contributing to inter tissue coordination of energy homeostasis (Jois et al., 2017).

### Adipose ChREBP Links Lipogenesis to Insulin Sensitivity

Impaired insulin signaling in adipose tissue is a critical feature of insulin resistance. Studies have reported that ChREBP activation in white adipose tissue can improve metabolic homeostasis by producing protective circulating signals (Yore et al., 2014; Tang et al., 2016). A class of mammalian lipids characterized by a branched ester linkage between a fatty acid and a hydroxy-fatty acid, palmitic acid hydroxyl stearic acid (PAHSA), was reported to exert beneficial effects on glucose homeostasis through direct and incretin-mediated modulation of β cell function, glucose uptake and reduction in inflammation (Yore et al., 2014). Similarly, adipose-specific ChREBP knockout (ChREBP^adiposeKO^), which exhibit low lipogenesis rates in adipose tissue, are insulin resistant with impaired insulin action in liver, muscle and white adipose tissue under both chow and high fat diet conditions. ChREBP^adiposeKO^ mice have lower serum levels of PAHSAs, while PAHSA supplementation, in particular the 9-PAHSA isomer, rescues ChREBP^adiposeKO^ global insulin resistance and adipose tissue inflammation, confirming that loss of adipose-ChREBP is sufficient to cause insulin resistance (Vijayakumar et al., 2017). A recent study identified the mechanistic target of rapamycin complex 2 (mTORC2) as a novel regulator of ChREBP (especially the β isoform) in adipose cells. Specific ablation of rapamycin-insensitive companion of mTOR (Rictor) in mature adipocytes impaired insulin-stimulated glucose uptake in adipose tissue leading to the down-regulation of ChREBPβ and target gene expression involved in lipogenesis control (Tang et al., 2016). In agreement with an important adipose–liver crosstalk mediated by ChREBP, these effects are associated with hepatic insulin resistance and enhanced gluconeogenesis. Altogether, these studies support an important role for adipose ChREBP in triggering insulin–sensitive signals (Tang et al., 2016).

### Novel Interaction Between Hormone-Sensitive Lipase and ChREBP in Adipose Tissue

ChREBP was recently identified as a partner of the lipolytic enzyme hormone-sensitive lipase (HSL) in adipose tissue (Morigny et al., 2019). Knockdown of HSL in human adipocytes and mouse adipose tissue was shown to enhance insulin sensitivity and induce the elongation of very long chain fatty acid enzyme (Elovl6). Elov16 is a microsomal enzyme that regulates the elongation of C12-16 saturated and monounsaturated fatty acids in a ChREBP-dependent manner (Morigny et al., 2019). At the mechanistic level, physical interaction between HSL and ChREBP impaired the nuclear translocation of ChREBPα and the subsequent induction of ChREBPβ and target genes, in particular Elovl6 (Morigny et al., 2019). This study reveals a novel regulation for ChREBP in adipose tissue. Inhibiting the interaction between HSL and ChREBP may lead to potential therapeutic strategies for improving insulin sensitivity in fat cells.

## Conclusion and Future Directions

ChREBP is now a well-established carbohydrate sensor. Although most studies have been dedicated to its implication in the control of the glycolytic and lipogenic pathways, recent data have also unraveled novel contributions of ChREBP in hepatocytes and in fat cells where it could be instrumental in producing hepatokines and/or lipokines triggering inter-organ crosstalk. As discussed, newly identified co-factors (epigenetic modifiers) and/or partners (adipose HSL) in these tissues may also represent potential therapeutic strategies for NAFLD and/or for improving systemic insulin sensitivity. Recent studies have also supported the importance of ChREBP in the regulation of fructose metabolism and have underscored the need for a better understanding of its role and regulation in the small intestine. Lastly, identifying specific and/or overlapping targets of ChREBPα and ChREBPβ in key cell types as well as determining their specific impact on insulin sensitivity will be of particular importance in the coming years.

## Author Contributions

All authors listed have made a substantial, direct and intellectual contribution to the work, and approved it for publication.

## Conflict of Interest Statement

The authors declare that the research was conducted in the absence of any commercial or financial relationships that could be construed as a potential conflict of interest.
